# Retro-dimension-cue benefit in visual working memory

**DOI:** 10.1038/srep35573

**Published:** 2016-10-24

**Authors:** Chaoxiong Ye, Zhonghua Hu, Tapani Ristaniemi, Maria Gendron, Qiang Liu

**Affiliations:** 1 Research Center of Brain and Cognitive Neuroscience, Liaoning Normal University, Dalian, 116029, China; 2 Department of Computer Science and Information Systems, University of Jyväskylä, Jyväskylä, 40014, Finland; 3 Department of Mathematical Information Technology, University of Jyväskylä, Jyväskylä, 40014, Finland; 4 Interdisciplinary Affective Science Laboratory, Northeastern University, Boston, MA, 02115, USA; 5 Key Laboratory for NeuroInformation of Ministry of Education, University of Electronic Science and Technology of China, Chengdu, 610054, China

## Abstract

In visual working memory (VWM) tasks, participants’ performance can be improved by a retro-object-cue. However, previous studies have not investigated whether participants’ performance can also be improved by a retro-dimension-cue. Three experiments investigated this issue. We used a recall task with a retro-dimension-cue in all experiments. In Experiment 1, we found benefits from retro-dimension-cues compared to neutral cues. This retro-dimension-cue benefit is reflected in an increased probability of reporting the target, but not in the probability of reporting the non-target, as well as increased precision with which this item is remembered. Experiment 2 replicated the retro-dimension-cue benefit and showed that the length of the blank interval after the cue disappeared did not influence recall performance. Experiment 3 replicated the results of Experiment 2 with a lower memory load. Our studies provide evidence that there is a robust retro-dimension-cue benefit in VWM. Participants can use internal attention to flexibly allocate cognitive resources to a particular dimension of memory representations. The results also support the feature-based storing hypothesis.

Visual working memory (VWM) provides online storage for visual information transferred from perceptual processing; this allows people to act on visual information that is no longer present in the environment^1^. Although VWM is flexible and goal oriented, it can only represent limited information from the total sensory input. Therefore, attention must be paid to ensure that only the most relevant visual information is encoded and maintained. Based on the targets of attention, attention can be classified as external attention and internal attention^2^. The former refers to the selection and modulation of sensory information, and the latter refers to the selection, modulation, and maintenance of internally generated information. In recent years, a number of studies have examined the influence of internal attention on VWM.

Griffin and Nobre^3^ investigated whether it is possible to orient selective spatial attention to internal representations held in VWM. They used a change detection task, which asked participants to remember four colors. Some participants received a retro-cue, which oriented them to a spatial location in working memory after the stimulus array disappeared. They found that, compared to a non-cue condition, there was a stable benefit in task performance from the presence of a retro-cue (also see Landman, *et al*.^4^). These results suggest that, even when the visual stimulus and iconic memory are gone, directing internal attention can still influence VWM representations.

Researchers put forward a variety of explanations of the mechanisms of the retro-cue benefit. Some researchers suggested that the performance for a cued item is improved due to an enhancement or strengthening of the representation of the cued item. As a result, the cued item suffers from less competition from the non-cued items in VWM, and this leads to faster and more accurate retrieval of the cued item. According to this assumption, non-cued items are maintained in VWM unchanged, but they are less accessible than the cued item^5^,^6^,^7^,^8^,^9^. Other researchers suggested that retro-cue can help to reduce memory load by removing non-cued items from VWM, thus the participant would have more free VWM resources to maintain the cued item^9^,^10^,^11^. Another suggestion is that attention is oriented to a particular memorized item, which makes VWM more resistant to visual interference from the test probe^12^,^13^,^14^,^15^. In addition, there are a series of studies suggesting a third memory stage, termed fragile visual short-term memory (FM), seems to exist between the iconic memory and robust VWM. FM has a large capacity (at least 2 items more than VWM) and long-lasting lifetime, it can exist almost as long as VWM without interference of new stimulation input. Thus, another account of the retro-cue benefit is that it arises because the participants selectively transfer the cued item from FM to robust VWM^4^,^16^,^17^,^18^,^19^. Finally, other researchers suggested that memory representations become degraded during the retention interval, and participants can use attention to protect the cued item from this degradation, or refresh the cued item to improve retrieval^20^,^21^,^22^,^23^. Of course, these explanations of the retro-cue benefit may not be mutually exclusive. For instance, strengthening and removal are not necessarily incompatible processes. It is possible that a cued item is strengthened and non-cued items are removed at the same time^9^,^24^.

To our knowledge, in previous studies using the retro-cue task, three main types of retro-cues were used. The first type of retro-cue is endogenous cue, which is presented at the center of the screen and points to a target location^3^,^4^,^7^,^8^,^9^,^11^,^13^,^15^,^17^,^18^,^19^,^20^,^22^,^25^,^26^,^27^,^28^,^29^,^30^,^31^,^32^,^33^,^34^,^35^,^36^,^37^,^38^,^39^,^40^,^41^,^42^,^43^,^44^,^45^,^46^; the second type of retro-cue is exogenous cue, presented in the target location^9^,^12^,^19^,^29^,^31^,^43^,^47^,^48^,^49^; the third type of retro-cue is feature cue, which is presented at the center of the screen and cues participants to one feature of the previously presented items. For example, participants receive a red color cue, and then should direct attention to the shape of the red colored memory representation^33^,^43^,^44^,^45^,^50^,^51^. By using these three types of cues, participants can direct internal attention to a single representation in VWM, so that the allocation of attention resources can be managed to obtain a retro-cue benefit. We termed these three type of cues as the retro-object-cues. Thus, previous explanations of the retro-cue benefit were developed to account for how a retro-object-cue can improve VWM performance.

However, beyond selecting information in an object-based manner, attention can also be deployed in a dimension-based manner. For example, researchers have studied the impact of cuing attention to a dimension in an external attention task (i.e., in visual search). Müller, *et al*.^52^ found that a valid semantic pre cue of a dimension of upcoming targets could be used to improve the performance in a search task. Previous researchers proposed a dimension-weighting account to explain how participants could use external attention to select a particular dimension from visual information input to improve search performance^53^,^54^. The dimension-weighting account assumes that the pop-out is ultimately based on the saliency activity integrated across separate dimensional saliency maps. As the total amount of weight is limited, an increase of weight assigned to one dimensional map entails a reduction of the weight assigned to other dimensional maps. If the target dimension is known in advance, the saliency signals from the target dimension are amplified relative to signals from other dimensions, which could help to guide the allocation of external attention. In addition, recent research also investigated the impact of selecting a dimension of external attention on VWM encoding. They asked participants to remember one dimension of all objects, while ignoring other dimensions and found that a change of stored task-irrelevant dimension dramatically affects performance, which suggested that VWM encoding is an object-based process. That is, whenever participants use external attention to select one dimension into VWM, the other dimensions are also memorized automatically^55^.

To recap, prior studies of internal attention and VWM mainly investigated that the influence of object-based cuing on VWM representations. Whereas previous studies that employed dimension-based cuing mainly focused on selection in external attention tasks. To our knowledge, there are no studies exploring the influence of dimension-based cuing on internal attention to VWM representations^24^. This is critical since the impact of dimension-based internal attention may depend on a completely different mechanism than object-based internal attention. Thus, in the present study we offered participants a retro-dimension-cue in a VWM task to observe if there is a retro-dimension-cue benefit and to further explore its potential mechanism.

In addition, the present study can also address another basic issue in the VWM literature. That is, what is the format of memory representations^56^? Two hypotheses have been proposed. The object-based storing hypothesis suggests that a given VWM representation is structured as a set of monolithic object representations, such that additional feature information will be maintained “for free” after all features have been integrated into one memory unit^57^,^58^. The other hypothesis, called the feature-based storing hypothesis, suggests that the visual features such as colors and orientations are independent and stored separately from each other, such that objects have multiple feature levels of representation in VWM^59^. These hypotheses would predict different result patterns for the effect of a retro-dimension-cue on a VWM task. Based on the object-based storing hypothesis, because features are bound to an integrated object, participants cannot forget a task-irrelevant feature of an integrated object, or weight resources to a task-relevant features of an integrated object. As a result, an object-based storing hypothesis might expect that the retro-dimension-cue would not result in a benefit. However, based on the feature-based storing hypothesis, because different features are stored separately, participants can use the retro-dimension-cue to reduce memory load by forgetting task-irrelevant features or enhance the memory of task-relevant features. As a result, according to a feature-based storing hypothesis we might expect better performance when we present a valid retro-dimension-cue to participants. In some sense, the predicted effect from this view is similar to a previous study, which presented two encoding displays and participants are told which display is going to be tested and which can be dropped^60^. Therefore, the results of the present experiments testing for a retro-dimension-cue can weigh in on the debate between the object-based storing hypothesis and the feature-based storing hypothesis.

In the present study, we used a recall task which included a retro-dimension-cue condition. One advantage of using a recall task is that it allows us to use model fitting, with the swap model^61^, to separate the mnemonic parameters of guess rate, non-target reported rate and memory precision. If there is a retro-dimension-cue benefit, the improvement of behavioral performance may result from multiple sources: an increase in memory precision, a decline in non-target reported rate, or a decline in the guess rate. Therefore, we unpacked the potential sources of the retro-dimension-cue benefit by using the swap model in our recall task. Another advantage of the recall task is that it could help to minimize interference produced by presenting a probe stimulus in the location of the memory object^10^,^62^.

## Experiment 1

### Methods

We asked participants to perform a double dimension recall task, in which 50% of the trials contained a valid cue, and the remainder 50% of trials contained a neutral cue. We also used the swap model to calculate the probability of guessing, non-target reporting and memory precision in each condition. This allowed us to observe whether a benefit of the retro-dimension-cue existed by comparing these two conditions.

#### Participants

Twenty undergraduate students (18 females, 18–21 years old) were recruited from the participant pool at the Minnan Normal University and received course credit for their participation. All participants had normal or corrected-to-normal vision and no history of neurological problems. Written informed consent was provided by each participant prior to the experiment. The study conformed to the Declaration of Helsinki and was approved by the ethics committee of Liaoning Normal University.

#### Stimuli

Visual stimuli were generated colored bars (1.1° in length, 0.4° in height) presented on a gray background. The color and orientation of each memory stimulus were randomly selected from 360 possible colors (1–360, in 1 color step) and 180 possible angles (1–180, in 1 step). A palette of 360 colors was used. The RGB values were assigned to ensure all the presented colors were highly saturated. The RGB value n was assigned the value as follows:


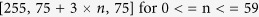



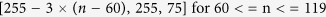



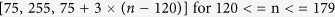



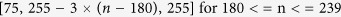



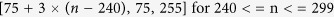



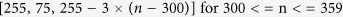


The bars could be presented in four possible locations, located at the corners of an imaginary square (eccentricity, 3°), with any two bars separated by at least 30 orientation degrees and 60 color steps. A fixation cross (0.2°) was presented in the center of the screen before the memory display onset. The valid cue stimuli were word “Color” (“
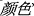
”, in Chinese), “Orientation” (“
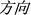
”, in Chinese) or neutral cue word “Random” (“
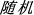
”, in Chinese) presented in black simsun-normal font (approximately 3.2° × 1.5°) at the center of the screen. The probe display of the color recall task consisted of an outlined square and a color wheel (5.8° inner radius; 2.2° thickness). The probe display of the orientation recall task consisted of an outlined square (1.2° × 1.2°) and a vertical white bar (1.1° × 0.4°, presented at fixation). The stimuli were presented on a 19” LCD monitor (1280 × 1024 pixel), and participants viewed the display at a distance of 60 cm in a dark room.

#### Task and design

**Figure 1:**
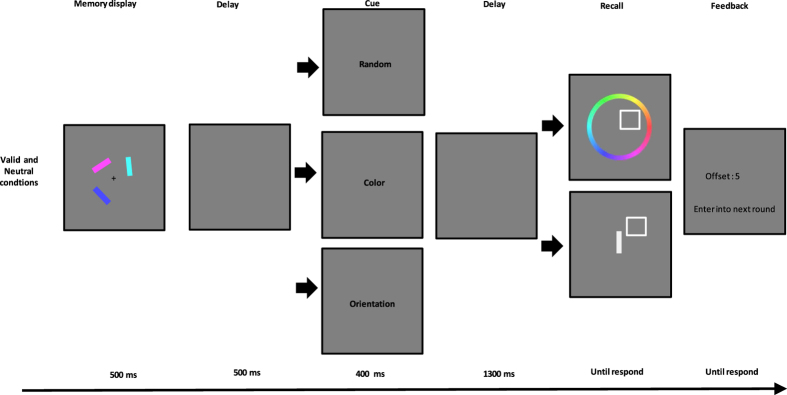
Trial structure of Experiment 1. In the neutral condition, the retro-cue was “Random” (top line); in the valid condition, the retro-cue was “Color” or “Orientation” (middle and bottom lines).

Participants were asked to perform double dimension recall tasks with the trial structures depicted in Fig. 1. Retro-dimension-cue type (valid, neutral) and report type (color, orientation) were manipulated within participants. Half of the trials included a retro-dimension-cue during the retention interval and these were randomly interleaved with the trials that included a neutral cue. All trials began with a fixation cross for 300 ms in the center of the screen. A memory display containing three colored bars was then presented for 500 ms. Participants were instructed to memorize both the color and orientation of the bars. After 500 ms had elapsed from the offset of the memory display, a dimension-cue (“Color” or “Orientation”, 100% valid) was presented at the center for a duration of 400 ms in the valid trials, and a neutral cue (“Random”) appeared for a duration of 400 ms in the neutral trials. The cue was then followed by the rest of the retention interval, which lasted 1300 ms. After the retention period, participants were asked to report on the color or orientation. The report type was selected at random on each trial. A white square outline appeared at the location of the probed VWM stimulus. For color report trials, a response wheel with invisible boundaries was centered on the fixation and consisted of 360 colored segments corresponding to possible stimulus colors. Participants were asked to report the color of the stored item at the location of white square outline. Participants selected one of 360 color values by clicking the left mouse button when the cursor was located in the desired value of the wheel. For orientation report trials, an adjustable vertical white bar was presented at fixation. Participants adjusted the white bar’s orientation to match that of the cued bar. Participants moved the cursor with the mouse; they pressed the left mouse button to rotate the white bar to the cursor position, and pressed the right mouse button to finalize their response when they were satisfied. Responses were not under time constraints. After the probe display disappeared, feedback on response error (in degrees) was provided. The next trial started 900–1100 ms after the feedback.

There were 100 trials for each condition (valid-color, neutral-color, valid-orientation, neutral-orientation), with a total of 400 trials. Trials were fully randomized. The task was split into 4 mini-blocks of 100 trials each, with a break of at least 15 s between mini-blocks. The entire experiment lasted approximately 60 min. Instructions at the beginning of each block informed participants of the task, and participants completed at least 16 practice trials before the main task.

#### Data Analysis

We computed the errors for each participant and each experimental condition (valid-color, neutral-color, valid-orientation, neutral-orientation) by subtracting the probed item’s value from the response. The main dependent variable was the absolute value of the deviation, which we called it offset. Then, we calculated a retro-dimension-cue benefit index (RDBI), which was defined as


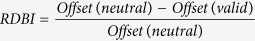


RDBI was thus the relative improvement between the valid and neutral conditions.

For each trial, we also calculated the errors in the reported color (or orientation) by subtracting the participant’s color (or orientation) setting from the memory color (or orientation). For model fitting, we fit the error data with the swap model using the MemToolbox^63^. This model assumes that participant’s behavior results from a mixture of three types of trials: On the first proportion of trials, participants hypothetically consolidated the items into VWM, which contains a noisy representation of the target color (or orientation), conformed to a von Mises distribution. On the second proportion of trials, participants hypothetically did not consolidate the items into VWM and simply guessed the reported color (or orientation) randomly, which should produce a uniform distribution. On the third proportion of trials, participants hypothetically reported the non-target color (or orientation) during the response phase, which is distribution of responses around non-target. For example, participants might report a non-target orientation on an orientation report trial or report a non-target color on a color report trial. This allowed us to estimate the guess rate (P_g_), the precision of the memory representation (SD) and non-target reported rate (P_b_) respectively. We fit the swap model to individual participant data in each condition.

#### Results

**Figure 2:**
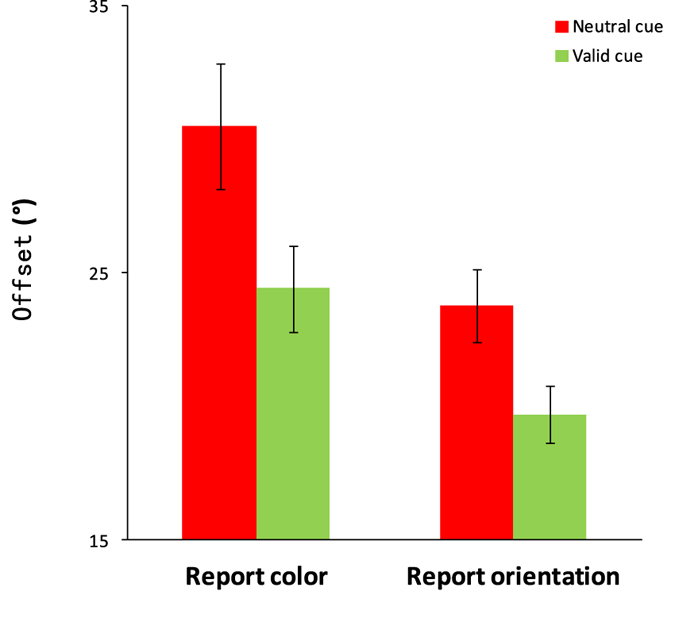
The offset results of Experiment 1. Error bars are standard errors of the mean.

The offsets were lower in the valid condition than in the neutral condition for both color report trials, t(19) = 5.402, p < 0.001, Cohen’s d = 2.48, and orientation report trials, t(19) = 6.017, p < 0.001, Cohen’s d = 2.76 (Fig. 2). The mean RDBI was 17.5% for color and 16.2% for orientation, which were both significantly greater than zero, t(19) = 6.258, p < 0.001, Cohen’s d = 2.87 (color), t(19) = 7.484,p < 0.001, Cohen’s d = 3.73 (orientation). The results demonstrate that the appearance of the retro-dimension-cue can lead to better performance.

**Figure 3:**
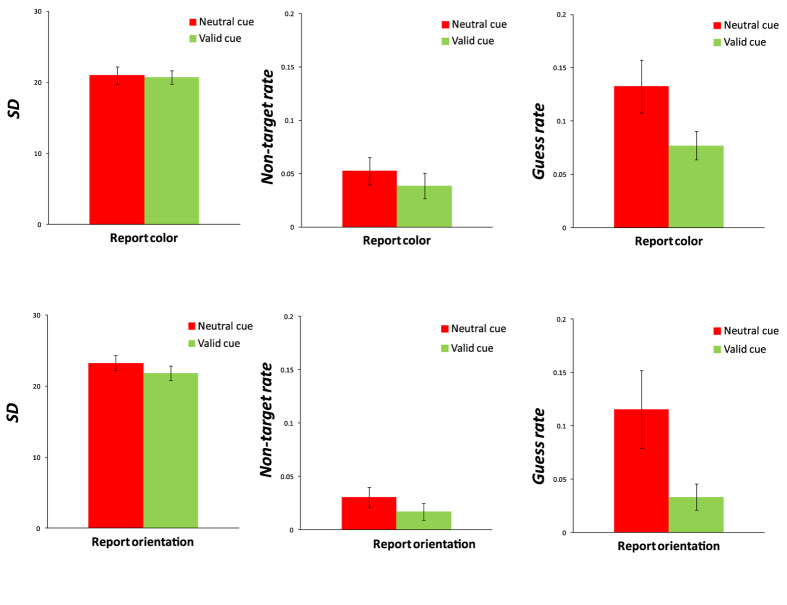
The results of Experiment 1 for memory precision (SD), non-target reported rate (P_b_), and guess rate (P_g_). The results of the report color (top line) and report orientation (bottom line) conditions are illustrated. Error bars are standard errors of the mean.

For the precision parameter (SD), there was no significant difference between the valid condition and the neutral condition for both color report trials, t(19) = 0.248, p = 0.807, Cohen’s d = 0.11, and orientation report trials, t(19) = 1.261, p = 0.223, Cohen’s d = 0.58. The Bayes factor analysis showed that the null hypothesis (i.e., no difference between the valid and neutral conditions) was 4.186 times (for reporting color) and 2.155 times (for reporting orientation) more likely to be true than the alternative hypothesis (a difference between conditions). For the non-target reported rate (P_b_), there was also no significant difference between the valid condition and neutral condition for both color report trials, t(19) = 1.131, p = 0.272, Cohen’s d = 0.52, and orientation report trials, t(19) = 1.244, p = 0.229, Cohen’s d = 0.58. The Bayes factor analysis showed that the null hypothesis was 2.457 times (for reporting color) and 2.194 times (for reporting orientation) more likely to be true than the alternative hypothesis. In contrast, the guess rates (P_g_) were significantly lower in the valid condition than in neutral condition, for both color report trials, t(19) = 2.206, p = 0.040, Cohen’s d = 1.01, and orientation report trials, t(19) = 2.383, p = 0.028, Cohen’s d = 1.09 (Fig. 3). In sum, the results showed that the appearance of the retro-dimension-cue led to a lower guess rate, but did not influence the non-target reported rate and memory precision.

#### Discussion

We asked participants to remember three colored bars, and found that a retro-dimension-cue could improve recall performance, indicating that there is a retro-dimension-cue benefit. In addition, we found that the source of the retro-dimension-cue benefit was not an increase in memory precision or a decline in non-target reported rate, but instead due to a decline in the guess rate. These results demonstrate that participants could use internal attention to select a dimension as target to improve the VWM representation of task-relevant dimension. However, as the object-based storing hypothesis would predict that the retro-dimension-cue could not cause a benefit of performance, these results do not provide evidence consistent with the object-based storing hypothesis of VWM.

One innovation of the present study was that we used a dimensional semantic word cue in a retro-cue task. Using cue words is a typical approach when researchers investigate whether knowledge of a dimension of an upcoming target will influence attention. For example, Müller, *et al*.^52^ investigated the issue by presenting a symbolic cue, the word “color”, “orientation” or “neutral”, to participants before a pop-out search task. Their results demonstrated that participants could use top-down control to bias their attentional weight to a task-relevant dimension based on the cue word. Further, there are a few retro-cue effect studies which presented a symbolic cue word as the retro-object-cue. Such as, the word “red “, “circle” or “wait” in Gilchrist, *et al*.’s^50^ study, and the word “red”, “green” or “all” in Hollingworth and Maxcey-Richard’s^33^ study. Our results showed that dimensional cue words can elicit a retro-dimension-cue benefit, consistent with prior work demonstrating that a semantic word cue is effective. Further, using semantic cue words provided an additional source of experimental control since it minimized the visual difference between valid and neutral cues (i.e., both were words).

One prior study has questioned whether object-based feature-cues are able to elicit a benefit. Berryhill, *et al*.^43^ found that a top-down retro-cue (a digit that mapped onto the location of one item) failed to evoke a retro-cue benefit. However, other research has demonstrated that object-based feature-cues can cause a retro-cue benefit^45^. In the present study, we also found that dimension-cues as a top-down retro-cue can benefit performance. One critical difference between Berryhill, *et al*.’s^43^ study and our own is the retention interval. The cue of our study was followed by a rest period of a 1300 ms (a retention interval) before the response period, whereas there was only a 400 ms retention interval between the cue and response in Berryhill, *et al*.’s^43^ study. Thus, it may take more time to use a retro-feature-cue to elicit a benefit. Even a spatial cue may require more than 400 ms to be effective, a recent study of van Moorselaar, *et al*.^39^ suggested that it takes about 500 to 600 ms to completely use an arrow cue to protect against perceptual interference. This could explain why we observed a benefit of retro-cue in our study, but no such benefit was observed in Berryhill, *et al*.’s^43^ study.

We also want to note that, importantly, in contrast to object-based feature-cues, dimension-based cues are abstract entities: In general feature-cues are used to describe target such as “the orientation of the *red* bar,” whereas statements such as “the orientation of a *colored* bar” used in dimension-cues do not convey any useful information to select one object. As a result, we need to be cautious about drawing conclusions from a dimension manipulation to other types of retro-cues, or from other types manipulation to retro-dimension-cues. There may be different mechanisms at play in feature-cue and dimension-cue experiments. As the retro-dimension-cue benefit is a new finding, Experiment 2 was conducted to try to replicate the results and further explore the mechanisms of retro-dimension-cue benefit.

## Experiment 2

There were two purposes in Experiment 2. The first purpose was to replicate the retro-dimension-cue benefit observed in Experiment 1. More importantly, the second purpose was to test whether the retro-dimension-cue benefit results from protection of representations of the cued dimension from degradation over time^20^,^21^. In other words, during maintenance, representations of the cued dimension might be unaffected by temporal decay that typically degrades representations of non-cued dimensions. The procedures of Experiment 2 were similar to those in Experiment 1, with the exception that we added short-delay neutral conditions to compare with the normal-delay neutral conditions. In the normal-delay neutral condition, the probe display appeared 1300 ms after the neutral cue (“Random”) disappeared. In the short-delay neutral condition, the probe display appeared only 50 ms after the neutral cue (“Random”) disappeared. This manipulation allows us to test whether there is a degradation effect in the VWM task. If there is a degradation effect, VWM performance will be better in the short-delay neutral condition than in the normal-delay neutral condition. On the contrary, if there is no difference of VWM performance between the normal-delay neutral and short-delay neutral conditions, this would demonstrate that there is no degradation effect in our experimental task. This would effectively rule out protection from degradation as a potential mechanism underlying the retro-dimension-cue benefit in our experimental task.

### Methods

#### Participants

Twenty-eight undergraduate students (26 females, 19–22 years old) were recruited from the participant pool at the Minnan Normal University and received course credit for their participation. All participants had normal or corrected-to-normal vision and no history of neurological problems. Written informed consent was provided by each participant prior to the experiment. The study conformed to the Declaration of Helsinki and was approved by the ethics committee of Liaoning Normal University.

#### Task and design

**Figure 4:**
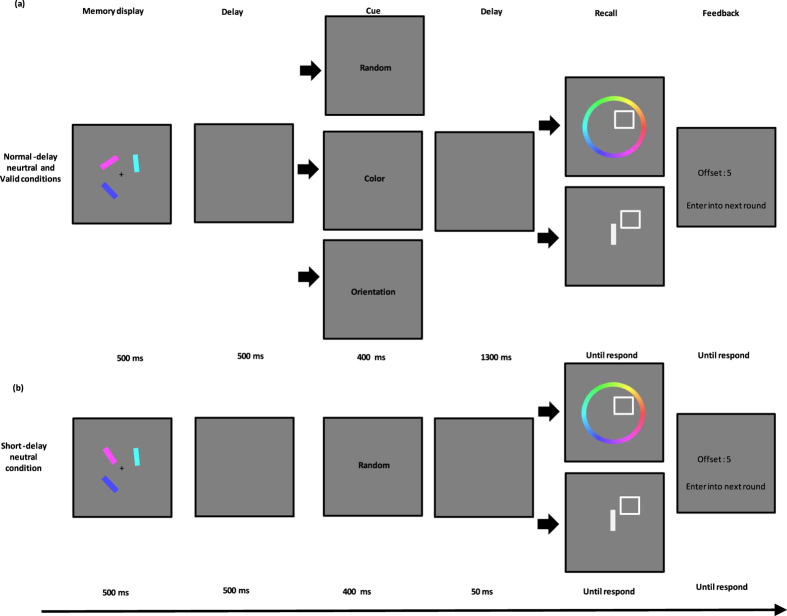
Trial structure of Experiment 2. (**a**) The normal-delay neutral and valid condition are illustrated. (**b**) The short-delay neutral condition is illustrated.

The design and procedure of Experiment 2 were identical to those of Experiment 1, except for the following changes: 1) Addition of two short-delay neutral conditions (one condition for color report, the other for orientation report). The procedure of short-delay neutral conditions in Experiment 2 was similar to neutral conditions in Experiment 1, except that the duration of the ISI between the neutral cue and the probe display was reduced from 1300 ms to 50 ms; 2) Adjusted number of trials. The number of trials in each condition (valid-color; short-delay neutral-color; neutral-color; valid-orientation; short-delay neutral-orientation; neutral-orientation) was reduced to 50. Thus, there was a total of 300 trials, which were fully randomized. The task was split into 3 mini-blocks of 100 trials each, with a break of at least 15 s between mini-blocks. The trial structures are depicted in Fig. 4. The entire experiment lasted approximately 50 min. Instructions at the beginning of each block informed participants of the task, and participants completed at least 16 practice trials before the main task.

#### Results

**Figure 5:**
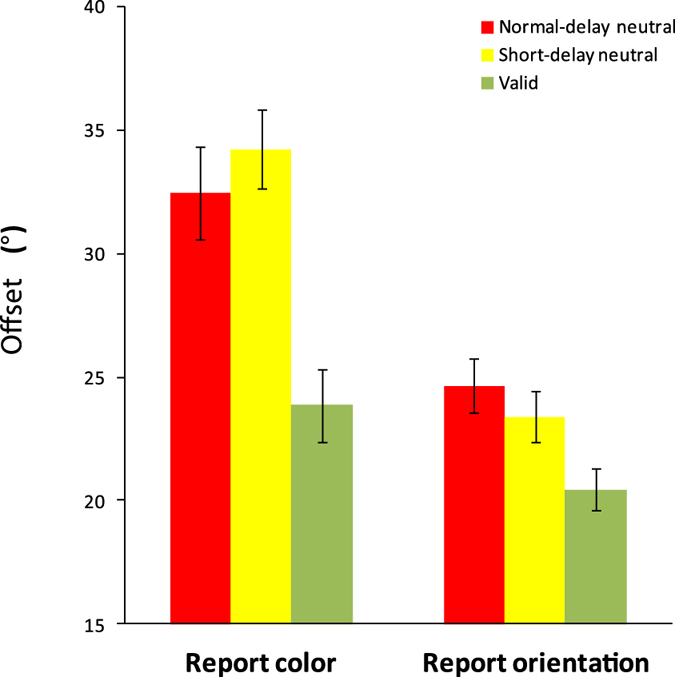
The offset results of Experiment 2. Error bars are standard errors of the mean.

For the offsets, one-way ANOVAs with cue condition as a factor (valid, short-delay neutral, normal-delay neutral) yielded a main effect for both color report trials, F(2,54) = 32.030, p < 0.001, η^2^ = 0.54, and orientation report trials, F(2,54) = 11.201, p < 0.001, η^2^ = 0.29. Post-hoc comparisons showed no significant difference in offsets between short-delay neutral and normal-delay neutral conditions for both color report trials, t(27) = 1.628, p = 0.115, Cohen’s d = 0.63, and orientation report trials, t(27) = 1.328, p = 0.195, Cohen’s d = 0.51. The Bayes factor analysis showed that the null hypothesis was 1.548 times (for reporting color) and 2.261 times (for reporting orientation) more likely to be true than the alternative hypothesis. The offsets were significantly lower in the valid condition than in the normal-delay neutral and short-delay neutral conditions for both color report trials, t(27) = 5.410, p < 0.001, Cohen’s d = 2.08 (normal-delay neutral vs. valid), t(27) = 7.188, p < 0.001, Cohen’s d = 2.77 (short-delay neutral vs. valid) and orientation report trials, t(27) = 4.482, p < 0.001, Cohen’s d = 1.73 (normal-delay neutral vs. valid), t(27) = 3.478, p = 0.002, Cohen’s d = 1.34 (short-delay neutral vs. valid) (Fig. 5). The results showed that the length of delay did not influence recall performance, and that the retro-dimension-cue led to better performance compared to both neutral conditions.

The mean RDBI was 23.6% for color and 15.1% for orientation, and both were significantly greater than zero, t(27) = 5.095, p < 0.001, Cohen’s d = 1.96 (color), t(27) = 4.680, p < 0.001, Cohen’s d = 1.80 (orientation). These results demonstrate a retro-dimension-cue benefit in Experiment 2.

#### Discussion

Experiment 2 replicated the observed benefit of a retro-dimension-cue. The experiment further suggested that after the disappearance of the neutral cue, the length of the blank interval did not influence recall performance. This finding indicates that memory representations did not become degraded during the retention interval in our task. Since we did not observe memory degradation due to the blank interval, the retro-dimension-cue benefit should not be simply interpreted as a protective mechanism against memory degradation.

## Experiment 3

Vogel and Awh^64^ used a change detection task to test 170 undergraduate students’ VWM capacity, and determined that the average memory capacity was 2.9. Thus, the memory set size of 3 in Experiment 1 and 2 could be considered a high VWM load set size for some participants. Lavie, *et al*.^65^ suggested an active mechanism of attentional control is needed for rejecting non-target information. But the capacity to engage in active control is expected to be weakened under high VWM load, resulting in increased processing of non-target information during the response phase. Consistent, our previous EEG study found that a non-reported dimension is computed automatically at a neural level before the response judgment^66^, such that computed outputs might interfere the response phase and negatively impact performance. Thus, when the memory load is potentially exceeding average capacity (i.e., Experiments 1 and 2), participants might suffer interference from processing of a non-reported dimension in the neutral condition, and the retro-dimension-cue can be used to reduce this interference by suppressing or removing non-reported dimension information in advance. If this is the only mechanism of retro-dimension-cue benefit, then when the memory load is lower, capacity available for rejecting information of non-reported dimension should be strengthened even in the neutral condition. This would lead to corresponding reduction of the retro-dimension-cue benefit. Thus we performed Experiment 3 with a reduced set size of items (down from three to two), and tested whether the retro-dimension-cue benefit disappeared when the set size was lower. If the benefit remained, we planned to further compare the RDBI of Experiment’s 2 and 3 to observe whether the degree of retro-dimension-cue benefit is impacted by the reduction of set size. We also reserved the short-delay neutral conditions, as in Experiment 2, to replicate the lack of degradation effect at low VWM load.

### Methods

#### Participants

Twenty-four undergraduate students (17 females, 18–21 years old) were recruited from the participant pool at the Minnan Normal University and received course credit for their participation. All participants had normal or corrected-to-normal vision and no history of neurological problems. Written informed consent was provided by each participant prior to the experiment. The study conformed to the Declaration of Helsinki and was approved by the ethics committee of Liaoning Normal University.

#### Task and design

The design and procedure of Experiment 3 were identical to those of Experiment 2, except that the total number of items in the memory display was reduced to two. On each trial, two colored bars were presented in two randomly chosen locations out of the four possible locations.

#### Results

**Figure 6:**
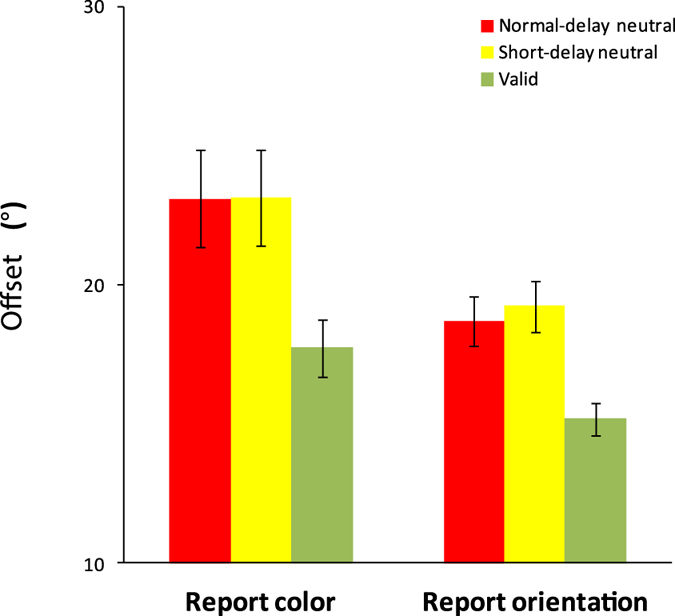
The offset results of Experiment 3. Error bars are standard errors of the mean.

For offsets, one-way ANOVAs with cue condition as a factor (valid, short-delay neutral, normal-delay neutral) yielded a main effect for both color report trials, F(2,46) = 8.548, p < 0.001, η^2^ = 0.27, and orientation report trials, F(2,46) = 20.189, p < 0.001, η^2^ = 0.54. Post-hoc comparisons showed no significant difference in offsets between the short-delay neutral condition and the normal-delay neutral condition for both color report trials, t(23) = 0.028, p = 0.978, Cohen’s d = 0.01, and orientation report trials, t(23) = 0.991, p = 0.332, Cohen’s d = 0.41. The Bayes factor analysis showed that the null hypothesis was 4.657 times (for reporting color) and 2.998 times (for reporting orientation) more likely to be true than the alternative hypothesis. The offsets were significantly smaller in the valid condition than in the normal-delay neutral and short-delay neutral conditions for both color report trials, t(23) = 3.312, p = 0.003, Cohen’s d = 1.38 (normal-delay neutral vs. valid), t(23) = 3.556, p = 0. 0.002, Cohen’s d = 1.48 (short-delay neutral vs. valid) and orientation report trials, t(23) = 4.589, p < 0.001, Cohen’s d = 1.91, (normal-delay neutral vs. valid), t(23) = 5.438, p < 0.001, Cohen’s d = 2.27 (short-delay neutral vs. valid) (Fig. 6). These results showed that the retro-dimension-cue benefit is observed when the memory load is low.

The mean RDBI was 16.5% for color and 16% for orientation, both were significantly higher than zero, t(23) = 2.686, p = 0.013, Cohen’s d = 1.12 (color), t(23) = 4.083, p < 0.001 (orientation), Cohen’s d = 1.70. In addition, we conducted a one-way ANOVA on the RDBI by treating the experiment as between-subject factor (Experiment 2 vs. Experiment 3). We found that the main effect of experiment was not significant for both color report trials, F(1,50) = 0.881, p = 0.352, η^2^ = 0.02, and orientation report trials, F(1,50) = 0.27, p = 0.87, η^2^ = 0.00. The results showed that the degree of retro-dimension-cue benefit in Experiment 3 was similar to the results of Experiment 2, such that no significant decrease in the retro-dimension-cue benefit was observed with the reduction of set size.

#### Discussion

In addition to replicating the null effect of retention interval, ruling out a degradation protection mechanism in our paradigm, the results of Experiment 3 showed that the retro-dimension-cue benefit was robust at a low VWM load, such that no reduction of the retro-dimension-cue benefit was observed compared to Experiment 2. These results demonstrated that the mechanism of the retro-dimension-cue benefit could not be simply be attributed to using the retro-cue to reduce interference from processing of a non-reported dimension during the response phase.

## General discussion

In Experiment 1, we found that performance was significantly better in the valid condition than in the neutral condition, and that the guess rate was significant lower in the valid conditions than in neutral condition, but there was no significant difference for non-target reported rate and memory precision between the valid and neutral conditions; in Experiments 2 and 3, when the set size was three and two, respectively, we found that there was no significant difference for behavior performance between the short-delay neutral and normal-delay neutral conditions, and performance was better in the valid condition than in both neutral conditions. Further, in these experiments, we found the same stable pattern of results regardless of whether participants were asked to report on color or orientation. Taken together, these findings indicate that participants can use a retro-dimension-cue to improve behavioral performance regarding a specified dimension during the maintenance process.

### Which mnemonic parameter is affected by the retro- dimension-cue?

In Experiment 1, we found that there was no difference in memory precision across conditions, but a lower guess rate in the valid condition than neutral condition. The swap model results showed that the improvement in performance as a function of retro-dimension-cue is reflected in an increased probability of reporting the target, but not on the probability of reporting the non-target and precision with which this item is remembered.

To our knowledge, this is the first study to use a recall task with a retro-dimension-cue. Previous studies showed that there is an inverse relationship between VWM number and memory precision^61^,^67^, so it follows that participants could use a spatial retro-cue to improve memory precision by removing the non-target representations. This is also confirmed by previous findings that a retro-object-cue benefit served to decline the guess rate and enhance the memory precision at the same time^10^,^12^,^39^. However, in our paradigm, participants could not use the retro-dimension-cue to decrease the number of items in VWM. Thus the mechanisms of a retro-object-cue may be completely different from the mechanisms of a retro-dimension-cue. Therefore, although we did not find the retro-dimension-cue improved memory precision, this finding is not conflict with the previous research demonstrating that retro-object-cues enhance memory precision.

Our findings may also appear to stand in contrast to Fougnie, *et al*.^68^. Fougnie, *et al*.^68^ used a recall task and asked participants to remember double dimension and single dimension representations, and found that there was no significant difference for guess rate between the double and single dimension condition, but the memory precision was higher in the single dimension condition than double dimension condition. We suggest that the different pattern of results between Fougnie, *et al*.’s^68^ study and our study is expected, because in Fougnie, *et al*.’s^68^ study, the task requirements were different between the double and single dimension conditions. As a result, participants were likely already processing the two conditions differently during the VWM consolidation phase. However, in our study, participants needed to remember both dimensions of memory items on each trial and the valid trials and neutral trials were randomly mixed. In each trial before the cue appeared, participants could not know if they will see a neutral cue or a valid cue, also could not know if they will be asked to report a color dimension or an orientation dimension. This experimental design might encourage participants to encode dimensions separately by only asking them to report a single dimension in each trial, but regardless of the encoding strategy, the cognitive processes before the cue appeared would be similar in the valid and neutral conditions.

Finally, a change in guess rate in our experiment, rather than precision, would be expected based on Bays, *et al*.^67^. Critically, Bays, *et al*.’s^67^ study demonstrates that a change in the precision of VWM representations occurs mainly in the early phase of encoding (i.e., the VWM consolidation phase) but not the VWM maintenance phase. Improving memory precision requires the perceiver to acquire new information from the visual stimulus, such that once the stimulus disappears, participants cannot enhance memory precision of representations any more. In our study, when the retro-dimension-cue appeared, the visual stimulus had already disappeared. Thus, participants did not have access to new visual information to improve memory precision. Therefore, the benefit of retro-dimension-cue is reflected in the stability of VWM representation, as demonstrated in the lower guess rates we observed.

### The mechanism of retro-dimension-cue benefit

We found that there were no performance differences between the short-delay neutral and normal-delay neutral conditions in Experiments 2 and 3. These results suggested that the length of the blank interval after cue disappearing did not influence recall performance. The lack of degradation effect is inconsistent with Pertzov, *et al*.’s^21^ study, which found a degradation effect in the retro-cue task. We think there are at least two reasons for this difference. First, Pertzov, *et al*.^21^ asked participants to remember four colored bars, but participants only need to remember two to three colored bars in our study. As Pertzov, *et al*.^21^ pointed out in their article, “multiple memory items may compete for memory resources and suppress each other’s representation leading to memory degradation”. Since we included fewer memory items, this should lead to less loss across time making it unlikely to observe a degradation effect in our Experiments 2 and 3. The second reason is that, In Pertzov, *et al*.’s^21^ study, behavioral performance was only slightly decreased from the 100 ms to the 1000 ms interval condition after the cue disappeared, but was significantly decreased from the 1000 ms to 3000 ms interval (for 100 ms interval, mean of errors is 11.8, SEM is 0.9; for 1000 ms interval, mean of errors is 13.9, SEM is 1.0; for 3000 ms interval, mean of errors is 18.4, SEM is 1.1). This is consistent with Zhang and Luck’s^69^ study which suggests that memory representations are not degraded during short intervals, but are suddenly degraded following long intervals. In our study, we use a 50 ms interval after the neutral cue disappeared as the short interval condition and 1300 ms interval as the long interval condition. Thus, even the longer interval condition is likely insufficient to elicit a degradation effect for remembering three colored bars. This reasoning can also explain why some other previous studies did not demonstrate a degradation effect. For example, van Moorselaar, *et al*.^39^ found that performance did not differ for a long-delay (1400 ms) non-cue condition and a short-delay (900 ms) non-cue condition, similar findings were also reported by Gressmann and Janczyk^70^. Because there is no degradation effect in our study, the mechanism underlying the retro-dimension-cue benefit is not simply due to protecting the representations of the cued dimension from degradation over time. In addition, we asked participants to remember two items in Experiment 3, which would not be a supracapacity set size for most participants, but we did not observe a reduction of the retro-dimension-cue benefit. This result implied that retro-dimension-cue benefit is also not simply due to protection from processing of non-reported dimension during the response phase. Thus, the retro-dimension-cue benefit could be caused by a combined mechanism of enhancing information about the reported dimension and removing information about the non-reported dimension.

We noted that the appearance of the probe display in neutral trials could give similar information to participants as a valid cue. For example, when the probe display included a color wheel in a neutral trial, the appearance of the probe display should cause an effect as giving participants a “Color” retro-cue. The appearance time of the probe display in the short-delay neutral condition is very close to the appearance time of a retro-cue in the valid condition. However, we observed that behavior performance was better in the valid condition than in short-delay neutral condition. Therefore, there may be different mechanisms between using a dimension cue (as the valid condition) and using a probe display (as the short-delay condition) to cue the target dimension. A similar result was observed in Souza, *et al*.’s^62^ study. They asked participants to perform a recall task with a retro-object-cue. In the no delay condition of their study, the cue and probe display appeared at the same time, 1000 ms after the visual stimulus offset. In the delay condition, the retro-cue appeared 1000 ms after the visual stimulus offset, and the probe display appeared 2000 ms after the visual stimulus offset. Although participants needed to maintain the VWM representations a longer interval in delay condition (1000 ms in the no delay condition, 2000 ms in the delay condition), they found that behavioral performance was better in the delay condition than in the non-delay condition. Their results implied that after participants received the cue, they needed some time to use attention to adjust cognitive resource allocation, otherwise the appearance of the probe display would interfere the resource reallocation process. Unlike the change detection task, in the recall task used here, there were no new visual items covering the location of memory items when the probe display appeared. Thus the interference caused by the probe display was not simply due to new visual input. Consistent with previous studies showing that cognitive demands of the test can also interfere with VWM representations during maintenance^71^, we suggest that the interference of a probe display in a recall task is caused by the cue and probe display appearing at the same time. This would result in the resource reallocation process, which is triggered by the cue, and the decision-making process, which is triggered by the probe display, competing for cognitive resources with each other, thus reducing performance on the recall task. Therefore, the appearance of the dimension-cue before the probe display can separate the resource reallocation process (dimension-weighting process) and decision-making process, avoiding cognitive interference from the probe display. This suggestion can explain why, in our Experiments 2 and 3, behavioral performance was much better in the valid condition than in the short-delay neutral condition. Thus, we also suggest that the retro-dimension-cue benefit was possibly caused by separating the resource reallocation process and the decision-making process. This makes our paradigm well suited for future researchers interested in exploring the mechanisms of the dimension-based selection of internal attention.

### Object-based encoding and feature-based storing

In the present study, our results supported the feature-based storing hypothesis and rejected the objected-based storing hypothesis. This is in line with Bays, *et al*.^72^ and Fougnie and Alvarez’s^73^ studies. In their studies, participants were asked to remember five to six double dimension items. The results showed a strong independence of errors between feature dimensions, suggesting participants could recall one feature accurately but forget the other feature of the same object, thus supporting the feature-based storing hypothesis. However, there are still some challenges to the feature-based storing hypothesis from recent studies. Marshall and Bays^74^ found that task-irrelevant dimensions were encoded into VWM automatically when participants were asked to store a task-relevant dimension. This finding suggests the alternative conclusion that VWM encoding is an object-based rather than feature-based process. In Bays, *et al*.^72^ and Fougnie and Alvarez’s^73^ studies, participants need to remember five to six double dimension items, as VWM capacity is limited, participants could not encode and maintain all items with perfect fidelity in their studies. Marshall and Bays^74^ suggested that the conflicting prior results of Bays, *et al*.^72^ and Fougnie and Alvarez’s^73^ study may be due to involuntary failure or variability in the encoding process for each dimension, resulting in independent errors on recall. However, this explanation suggested by Marshall and Bays^74^ does not account for the findings of our Experiment 3. In Experiment 3, participants only needed to remember two items, which could be encoded and maintained perfectly, according to previous research^57^. As a result, there should be little to no failure or variability in encoding for each dimension in our data. Yet we still observe results which are consistent with VWM storage at the feature level. Therefore, our study provides new evidence to support that view that VWM representations can be stored in a feature-based manner.

To be clear, our study does not challenge Marshall and Bays’s^74^ findings, and we have noted that their findings are also consistent with other studies^75^. Instead, we propose that participants encoded the memory items in an object-based manner involuntarily, but could store them in VWM in a feature-based manner voluntary. This explanation that could integrate both sets of previous findings, based on object-based encoding and feature-based storing. As we know, there may be independent mechanisms for the consolidation and maintenance of information in VWM^76^, and we suggest that memory encoding and memory maintenance have different processing mechanisms. During the memory encoding phase (VWM consolidation), participants could process the VWM representation in an object-based way, such that task-irrelevant information of items would consolidate into VWM involuntarily. After the encoding phase is completed, the unit of VWM representations will become more detailed during the VWM maintenance process. VWM representations could be independently stored at the feature level, and participants could reallocate cognitive resources voluntarily to one given dimension according to task requirements. This explanation is also supported by Xu’s^77^ fMRI study, which found that participants initially encode memory items in an object-based manner, but gradually prune the task-irrelevant features during the VWM maintenance phase. In addition, Vergauwe and Cowan’s^78^ recent study showed that participants can flexibly store VWM representations as integrated objects or as independent features, according to task requirements. Although our study provides new evidence for feature-based storing hypothesis, this does not preclude the possibility that participants could store VWM representations in an object-based manner. Thus, we believe that in the future researchers could consider both object-based and feature-based mechanisms in VWM as not mutually exclusive, and potentially compatible. Our paradigm could be used to further explore object-based encoding and feature-based storage.

## Summary and Conclusion

In summary, our results show a stable retro-dimension-cue benefit in VWM. These results demonstrated that participants can use internal attention to flexibly allocate cognitive resources to a particular dimension of VWM. We reject the possibility that the benefit is only caused by protection from degradation, or reducing the interference from processing of a non-reported dimension during the response phase. Our results further support the notion that visual features could be independent and stored separately from each other, such that objects have multiple feature levels of representation in VWM.

## Additional Information

**How to cite this article**: Ye, C. *et al*. Retro-dimension-cue benefit in visual working memory. *Sci. Rep*. **6**, 35573; doi: 10.1038/srep35573 (2016).
